# Value of the 8-oxodG/dG ratio in chronic liver inflammation of patients with hepatocellular carcinoma

**DOI:** 10.1016/j.redox.2016.02.003

**Published:** 2016-02-09

**Authors:** Pengcheng Li, Grant A Ramm, Graeme A Macdonald

**Affiliations:** a The Medical School, The University of Queensland, QLD 4006, Australia; b Hepatic Fibrosis, QIMR Berghofer Medical Research Institute, QLD 4029, Australia; c Department of Gastroenterology and Hepatology, Princess Alexandra Hospital, QLD 4102, Australia

**Keywords:** Hepatocellular carcinoma, 8-oxodG, Chronic liver inflammation, Oxidative DNA damage, HPLC-MS/MS, Hepatocarcinogenesis

## Abstract

The aim of this study was to examine the role of oxidative DNA damage in chronic liver inflammation in the evolution of hepatocellular carcinoma. The accumulated data demonstrated that oxidative DNA damage and chronic liver inflammation are involved in the transformation of normal hepatocytes and their evolution towards hepatocellular carcinoma. However, the levels of 8-oxy-2′-deoxy-guanosine (8-oxodG), a biomarker of oxidative DNA damage, were overestimated and underestimated in previous reports due to various technical limitations. The current techniques are not suitable to analyze the 8-oxodG levels in the non-malignant liver tissues and tumors of hepatocellular carcinoma patients unless they are modified. Therefore, in this study, the protocols for extraction and hydrolysis of DNA were optimized using 54 samples from hepatocellular carcinoma patients with various risk factors, and the 8-oxodG and 2′-deoxyguanosine (dG) levels were measured. The patients enrolled in the study include 23 from The Princess Alexandra Hospital and The Royal Brisbane and Women's Hospitals, Brisbane, Australia, and 31 from South Africa. This study revealed that the 8-oxodG/dG ratios tended to be higher in most non-malignant liver tissues compared to hepatocellular carcinoma tissue (*p*=0.2887). It also appeared that the ratio was higher in non-malignant liver tissue from Southern African patients (*p*=0.0479), but there was no difference in the 8-oxodG/dG ratios between non-malignant liver tissues and tumors of Australian hepatocellular carcinoma patients (*p*=0.7722). Additionally, this study also revealed a trend for a higher 8-oxodG/dG ratio in non-malignant liver tissues compared to tumoural tissues of patients with HBV. Significant differences were not observed in the 8-oxodG/dG ratios between non-cirrhotic and cirrhotic non-malignant liver tissues.

## Highlights

• Higher ratios of 8-oxodG/dG in most non-malignant liver tissues than HCC tissues.
• Higher ratio in the non-malignant liver tissues than tumors of patients with HBV.
• No significant differences of ratios of 8-oxodG/dG between no-cirrhosis and cirrhosis.
• A suitable method for 8-oxodG measurement from human liver tissues.

## Graphical abstract

**Figure:**
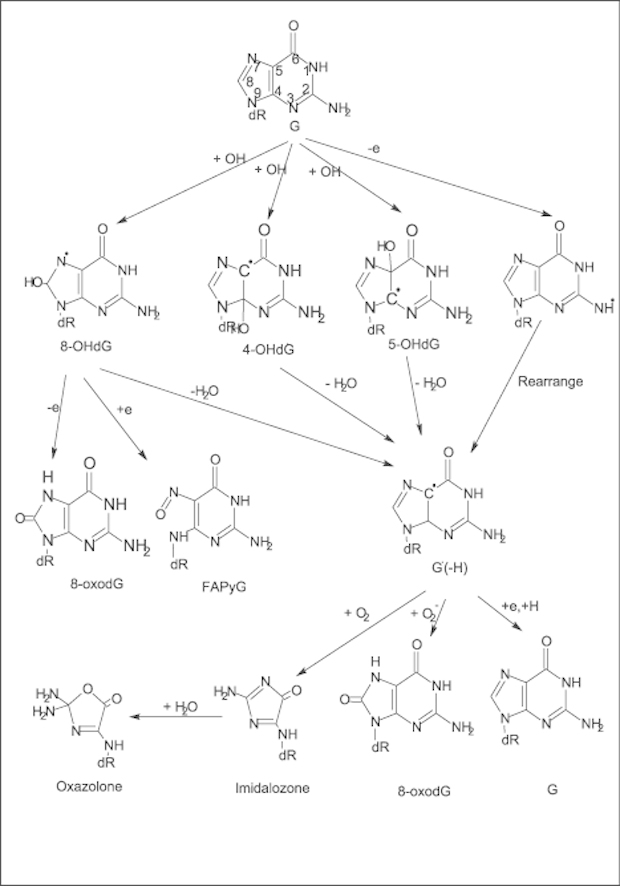

## 1 Introduction

The mechanisms of hepatocarcinogenesis are incompletely understood [1], [2], [3]. Mounting evidence indicates that oxidative DNA damage caused by reactive oxygen species (ROS) and reactive nitrogen species (RNS) accumulation in chronic liver inflammation may play a role in hepatocarcinogenesis [4], [5]. A number of investigators have suggested that hepatocellular carcinoma (HCC) develops from the malignant transformation of hepatocytes, as these cells acquire multiple ROS- and RNS-induced mutations in key genes that control cell proliferation and death [6], [7]. This assumption has been supported by several observations. The prevalence of chromosomal gene alterations increases with the progression from chronic hepatitis to fibrosis, cirrhosis, low grade dysplasia, high grade dysplasia, early HCC, moderately differentiated HCC and finally advanced HCC [8], [9]. The epidemiological data demonstrate that there is a strong relationship between chronic liver inflammation and hepatocarcinogenesis [10], [11], [12]. Approximately 80% of HCC cases are associated with chronic liver inflammation and liver cirrhosis [13], [14]. Chronic liver inflammation may produce ROS [15], [16] and RNS [17], [18]. ROS and RNS may cause DNA oxidation, nitrosylation, nitration, and halogenation, leading to mutations in key genes, including oncogenes and tumoural suppressor genes [7], [19], [20]. These mutations likely confer growth advantages on these cells, leading to the transformation of normal hepatocytes and their evolution towards HCC.

As mentioned in previous articles, 8-oxodG is one of the main oxidation products of guanosine (dG) and is induced by ROS and RNS [19], [21], [22]. The presence of 8-oxodG in DNA leads to misreading and misinsertion of nucleotides during DNA synthesis, leading to G→T and G→C conversions [23], [24], [25]. 8-oxodG can be produced from continuous oxidative stresses associated with chronic inflammation [21]. A previous study revealed that the 8-oxodG levels are elevated in some human pre-neoplastic lesions and cancerous tissues [26]. Moreover, 8-oxodG has also extensively been used as an indicator of oxidative DNA damage [27] and various diseases [28], [29], [30].

The picture is less clear for hepatocarcinogenesis. Furthermore, it has not been completely confirmed whether the genetic alterations induced by oxidative DNA damage are involved in the malignant transformation of normal hepatocytes [31]. Currently, one issue with the data is the concern about whether the artefactual production of 8-oxodG during DNA processing (DNA extraction, hydrolysis and analysis) leads to an over- or underestimation of the role of 8-oxodG in malignant transformation [32], [33], [34]. In the past, the accurate measurement of 8-oxodG in samples of human liver tissue has been hampered by limitations in the amount of tissue available for study, the incomplete release of nucleosides, the artefactual formation of 8-oxodG during tissue processing, and the limits of detection of the assays employed to measure the 8-oxodG levels [27]. The European Standards Committee on Oxidative DNA Damage (ESCODD) and other investigators have attempted to develop reliable protocols for sample preparation and analysis, with minimal dG oxidation as a consequence of sample preparation [27], [35], [36], [37], [38], [39]. However, both overestimation and underestimation of 8-oxodG concentrations have been reported by ESCODD using various methods [37]. The conclusion reached from reviewing the previous studies, including those from ESCODD, is that the current techniques are not suitable to analyze the 8-oxodG levels in non-malignant liver tissues and tumors of HCC patients unless they are modified.

Therefore, in this study, the protocols for extracting and hydrolyzing patients' DNAs were optimized, and the 8-oxodG levels were measured. This study evaluated the dose-dependent relationships between the amount of 8-oxodG (determined from the ratio of 8-oxodG/dG) and clinical variables in 54 patients with HCC, with particular attention paid to optimizing the experimental conditions to minimize the formation of 8-oxodG during the process. The aim of this study was to examine the role of oxidative DNA damage in the evolution of HCC by particularly focusing on oxidative DNA damage in chronic liver inflammation.

## 2 Materials

### 2.1 Samples

Frozen liver tissues from 54 HCC patients were studied in this experiment. These liver tissues were collected by medical doctors in hospitals in Australia and South Africa. Twenty-three patients from The Princess Alexandra Hospital and The Royal Brisbane Hospitals, Brisbane, Australia and 31 patients from South Africa were enrolled in this study. The Australian cases were drawn from a tissue bank, while the clinical material from South Africa was obtained from Professor Michael Kew. The details of each case are presented in Table 1. Both non-malignant liver tissues and HCC tissues were available for some cases, while only non-malignant liver tissues or HCC tissues were available from other cases. In addition, the clinical data were not complete for all cases; therefore, the data on the presence or absence of cirrhosis or on the risk factors for chronic liver disease were not available for some cases. These studies were approved by the Human Research Ethics Committee of the Royal Brisbane Hospital and the University of Queensland.

## 3 Methods

### 3.1 Reagents

Zinc chloride, magnesium chloride, calcium chloride, sodium chloride, sodium acetate, guanidine thiocyanate, 2,2,6,6-tetramethylpiperidine-Noxyl (TEMPO), chloroform, 2-propanol, isoamyl alcohol, nuclease P1_,_ proteinase K, RNase A, alkaline phosphatase, catalase and Tween 20 were purchased from Sigma (St. Louis, MO, USA). The Phase Lock Gel (PLG) tubes were purchased from Eppendorf-Netheler-Hinz (Hamburg, Germany). Tris base was purchased from Amresco (Solon, OH, USA).

### 3.2 Equipment

High Performance Liquid Chromatography – tandem mass spectrometry was performed with a PE/Sciex API 300 mass spectrometer equipped with a turbo-ion spray interface coupled to a Perkin Elmer series 200 HPLC system (Waltham, MA, USA) from Queensland Health Scientific Services (39 Kessels Rd, Coopers Plains Qld 4108, Au).

### 3.3 Preparation of the homogenization buffer and enzyme buffer

Homogenization buffer was prepared with 20 mM Tris, 5 mM magnesium chloride, 50 U/ml catalase and 1 mM TEMPO and then adjusted to pH 7.5. Tween 20 was dissolved in homogenization buffer to a final concentration of 0.5% Tween 20 (v/v). Guanidine thiocyanate (GTC) was dissolved in Milli-Q water to produce a 4 M GTC DNA extraction solution containing 4 M GTC, 50 U/ml catalase and 1 mM TEMPO. One volume of isoamyl alcohol was mixed with 24 volumes of chloroform to produce the Sevag solution, as previously described. Antioxidants (catalase, TEMPO) were added to all solutions, except 2-propanol and 70% v/v ethanol. Milli-Q water was used throughout. All solutions, except the Sevag solution, were stored in the dark at 4 °C in plastic bottles to avoid metal contamination from glass.

The RNase A buffer consisted of 100 µg/ml RNase A, 2 mM calcium chloride, and 20 mM Tris, pH 7.5. Proteinase K was added to 2 mM calcium chloride and 20 mM Tris buffer (pH 7.5) to prepare a 20 mg/ml proteinase K solution, which was stored at −20 °C. The hydrolysis buffer contained 25 mM sodium acetate and 0.1 mM zinc chloride. The pH value of the hydrolysis buffer was adjusted to 5.3, and it was then stored in a cold room. Nuclease P1 was dissolved in hydrolysis buffer containing 50 mM sodium acetate and 0.2 mM zinc chloride to produce a 2.5 µg/µl solution. This solution was divided into small aliquots and stored at −20 °C. The alkaline phosphatase solution and catalase were stored at 4 °C.

### 3.4 Comparison of the proteinase K digestion and 4 M GTC methods of DNA extraction

Previous studies have shown that proteinase K digestion at 37 °C generates more artefactual 8-oxodG than the cold 4 M GTC method at 0 °C. In this study, DNA from human liver tissues was isolated with these two methods to compare their effects on the efficiency of DNA extraction and the generation of 8-oxodG DNA so that the method that generated the least amount of artefactual 8-oxodG could be used for the subsequent isolation of DNA from human liver tissues.

Four frozen samples of human HCC tissues and four samples of normal human liver tissues were studied. The samples were distributed according to the method of extraction (Groups One and Two). Each group consisted of two HCC samples and two normal liver tissue samples. In a cold room, 50 mg of each tissue was homogenized with a high speed homogenizer. The homogenate was centrifuged at 1000*g* for 5 min. The supernatant was discarded, and the nuclear pellets were washed twice with Tween 20 buffer, followed by centrifugation at 1000*g* for 5 min after each wash. The nuclear pellets of Group One were used to isolate DNA by proteinase K digestion, whereas the Group Two samples were stored in the cold room to isolate DNA using the cold 4 M GTC method.

The nuclear pellets of each sample in Group one were dissolved in 540 µl of RNase A buffer and incubated in a 37 °C water bath for 30 min. Subsequently, 14 µl of proteinase K was added and incubated at 37 °C for 45 min. The solution was transferred to a prespun 2.0-ml PLG tube (heavy) and 560 µl of Sevag solution was added. The tubes were centrifuged at 13,000*g* for 5 min. This led to the formation of a mixed organic/aqueous solution in which the proteins and lipids precipitated in the organic phases in the PLG tubes and the DNA remained in upper aqueous phases. This supernatant was transferred to a 2-ml PLG tube (light), and then, an additional 560 µl of Sevag solution was added. These tubes were mixed and centrifuged at 13,000*g* for 5 min. The upper aqueous phase containing the DNA was transferred to a new 2-ml tube. Seventy-five microliters of a 5 M sodium chloride solution and 635 µl of isopropanol were added to each tube. After mixing, DNA was precipitated at −20 °C for 15 min and then centrifuged at 20,800*g* for 10 min. The supernatant was discarded, and DNA was stored at −80 °C prior to hydrolysis.

The crude nuclei of each sample in Group Two were completely dissolved in 850 µl of cold (0 °C) 4 M GTC solution in a cold room. The solution was transferred to a 2-ml PLG tube (heavy). Eight-hundred-fifty microliters of Sevag solution were added to this tube. The tube was centrifuged at 13,000*g*, and then, the upper phase containing the DNA was transferred to 2-ml PLG tube (light) before an additional 850 µl of Sevag solution was added. These tubes were mixed and centrifuged at 13,000*g* for 5 min. Then, the upper aqueous phase containing the DNA was transferred to a new 2-ml tube and 850 µl of 2-isopropanol was added and incubated at −20 °C for 15 min to precipitate the DNA. DNA was pelleted by centrifugation at 20,800*g* for 10 min, and the samples were stored at −80 °C prior to hydrolysis.

The DNA samples from Groups One and Two were hydrolyzed with 2 µg of nuclease P1 and 1 unit of alkaline phosphatase for 1 h at 50 °C for 1 h. The concentrations of 8-oxodG and dG were measured by HPLC-MS/MS.

### 3.5 Enzymatic hydrolysis of commercial calf thymus DNA

Accumulating data reveal that the ratios of 8-oxodG/dG vary in repeated measurements of the same samples from different individuals and in different laboratories [37], as well as those using different methods for sample preparation [27], [40] and different methods for detecting 8-oxodG [27]. These variations were up to several orders of magnitude. For example, the quantity of 8-oxodG from lymphocyte DNA was 4.24 per 10^6^ dG measured using HPLC, whereas it was 0.34 8-oxodG per 10^6^ dG measured using the comet assay [27]. Concentrations of 8-oxodG ranged from 2.23 to 441 8-oxodG per10^6^ dG in DNA from pig liver using HPLC techniques [40]. The inconsistency in the quantitation of the 8-oxodG/dG ratios implies that the actual amount of 8-oxodG in DNA cannot be determined as a result of the unsuitable hydrolysis conditions during processing. The release of 8-oxodG from DNA during enzymatic hydrolysis is influenced by a few factors, including the DNA concentration, choice of enzymes, enzymatic activities, incubation time and incubation temperatures. Excessive DNA, unsuitable enzymes, short incubation times and low temperatures may cause incomplete hydrolysis of DNA, while high temperature generates artefactual 8-oxodG. These drawbacks can result in an overestimation or underestimation of the 8-oxodG concentrations [32], [41]. Currently, most protocols of DNA hydrolysis are performed with approximately 100 µg of DNA, 1 to 20 µg of nuclease P1 (P1) and 0.5–20 U/ml of alkaline phosphatase for a few minutes to hours at 37 °C or overnight in a cold room [27], [41], [42]. However, 100 µg of DNA are not completely hydrolyzed by 1 U/ml of nuclease P1 during a 1.5 h incubation hours at 37 °C, followed by a 1 h incubation at 37 °C with 1 U/ml of alkaline phosphatase, even if the doses of nuclease P1 or alkaline phosphatase are increased [41]. Nuclease P1 and alkaline phosphatase can more rapidly and efficiently hydrolyze DNA at high temperatures, such as 65 °C, compared to 37 °C, but incubation periods in excess of 15 min at 65 °C increase the levels of artefactual 8-oxodG [43]. The denaturation of DNA into single stands by incubating it at high temperatures is beneficial for complete hydrolysis [41], but the 100 °C temperature used to denature DNA may increase the levels of artefactual 8-oxodG. Another study shows that 100 µg of DNA plus was completely hydrolyzed by 1 µg P1 and 1 U/ml alkaline phosphatase in 1 h at 50 °C and produced less artefactual 8-oxodG [32].

Therefore, in this study, five different hydrolysis conditions were used to compare the generation of 8-oxodG in calf thymus DNA. Based on these data, suitable hydrolysis conditions were optimized for the subsequent hydrolysis of the DNA from human liver tissues.

One-hundred micrograms of calf thymus DNA were dissolved in 90 µl of a hydrolysis solution and then hydrolyzed with 1 µg, 5 µg, 10 µg or 20 µg of nuclease P1 plus 1 unit of alkaline phosphatase using the following conditions:

i. The DNA solution was digested with nuclease P1 and alkaline phosphatase for 1 h at 50 °C.
ii. The DNA solution was first boiled at 100 °C for 5 min in a microwave oven and then rapidly chilled on ice for 2 min. Next, nuclease P1 and alkaline phosphatase were added to digest the DNA and incubated for 1 h at 50 °C.
iii. The DNA solution was incubated with nuclease P1 for 1 h at 50 °C before it was incubated with alkaline phosphatase for 1 h at 37 °C.
iv. The DNA solution was digested with nuclease P1 for 10 min at 65 °C and then treated with alkaline phosphatase for 1 h at 37 °C.
v. The DNA solution was digested with nuclease P1 for 30 min at 37 °C and then treated with alkaline phosphatase for 1 h at 37 °C.

After enzymatic hydrolysis, the solution was transferred into a 1.5-ml Phase Lock Gel (light) tube. One-hundred microliters of Sevag solution was added to each tube, and the tubes were briefly mixed, and centrifuged at 13,000*g* for 5 min. The proteins and Sevag solutions precipitated in the organic phase of the tube, while 8-oxodG, dG and DNA remained in the upper phases. The supernatant was transferred to a new 0.5-ml tube and stored at −80 °C before the 8-oxodG levels were measured by HPLC-MS/MS.

### 3.6 DNA extraction from the human liver tissues using the 4 M GTC method

The DNA from patients' liver tissues was isolated using the cold 4 M GTC method. All procedures were performed in a cold room, unless stated otherwise. The dissection materials, reagents, 10-ml flat-bottom tubes and equipment used for DNA extraction were pre-chilled. Approximately 50 mg of frozen liver tissue per sample was cut on aluminum foil on dry ice, weighed, and immediately placed into numbered 10-ml flat-bottom tubes. One milliliter of ice-cold homogenization buffer was added to each numbered 10-ml tube on ice. The samples were completely homogenized with a power homogenizer for three minutes. After each sample was homogenized, the pestle was sequentially washed in pre-chilled 100% alcohol, Milli Q water and 100% alcohol, and then dried using a Kimwipe tissue.

The homogenized solution for each sample was transferred from the 10-ml flat-bottom tube into a marked 2-ml centrifuge tube. One milliliter of homogenization buffer was added to the 10-ml flat-bottom tube to wash the tube and then added to the 2-ml tube. The solution was mixed with a vortex mixer for 1 min and then placed on ice for 5 min before the crude nuclei were pelleted by centrifugation at 1000*g* for 10 min. After discarding the supernatant, which contains membranes, proteins, mitochondria and most of the RNA, the nuclear fraction was re-suspended in one ml of Tween 20 buffer and placed on ice for 5 min. The samples were centrifuged at 1000*g* for 10 min, the supernatant was withdrawn, and the nuclei were re-suspended in one ml of Tween 20 buffer and placed on ice for 5 min. The samples were centrifuged a final time at 1000*g* for 10 min, and the supernatants were discarded

The pellets were dissolved in 850 µl of cold 4 M GTC by pipetting up and down to produce a clear solution and then incubated on ice for 20 min. After the pellets were completely dissolved, the DNA solution was transferred to a 2.0-ml pre-spun PLG (heavy) tube. Eight-hundred-fifty microliters of cold Sevag solution was added to this tube, which was shaken by hand for 1 min, and then centrifuged at 13,000*g* for 5 min. On completion, the supernatant containing the DNA was transferred into a 2-ml tube and 850 µl of cold isopropanol were added and incubated at −20 °C for 1 h to precipitate the DNA. The solution was centrifuged at 16,000*g* for 10 min at 4 °C, and the supernatant was discarded. The pellets were re-suspended in 800 µl of cold 70% v/v ethanol and centrifuged for 3 min at 16,100*g* at 4 °C. The supernatant was carefully discarded before the tubes were drained by inversion on absorbent paper. One-hundred microliters of pre-chilled hydrolysis buffer was added to each tube to completely re-suspend the DNA.

### 3.7 Measuring the DNA concentrations

The concentrations of the DNAs isolated from the patients’ liver tissues were measured using a spectrophotometer. Two microliters of the DNA suspension was mixed with 98 µl of Milli Q water. The absorbance of the resulting solution was measured at A260 nm and the DNA concentration was calculated.

### 3.8 DNA hydrolysis

Based on the optimization of the hydrolysis conditions, 2 µg of nuclease P1 and one unit of alkaline phosphatase were added to each tube and then mixed. The solution was incubated for 1 h at 50 °C. After incubation, this solution was transferred into a 0.5 ml pre-spun PLG (light) tube, 100 µl of Sevag solution was added and the tube was briefly mixed before being centrifuged at 13,000*g* for 5 min. After centrifugation, the supernatant solution containing hydrolyzed DNA was transferred to a fresh 0.5-ml tube and stored at −80 °C prior to the HPLC analysis.

### 3.9 HPLC-MS/MS analysis

The concentrations of 8-oxodG and dG were determined with HPLC-MS/MS by an expert in Queensland Health Scientific Services (39 Kessels Rd, Coopers Plains Qld 4108, Au). Separation was achieved using an Altima C18 column at 35 °C and a flow rate of 0.8 ml min^−1^, with a linear gradient starting at 100% A for 0.1 min, ramped to 80% B in 12 min, held for 2 min and then to 100% A for 1 min and equilibrated for 7 min. (A=1% methanol/deionized water, B=60% methanol/deionized water, both in 5 mM Ammonium Acetate). The dead space in the system modified the actual gradient at the column and was equivalent to approximately 3 min at 100% A before the start of the gradient. Under these conditions, the retention times for dG and 8-oxodG were 8.58 and 8.75 min, respectively (total run time 18 min). The column effluent was split to achieve a flow rate of 0.25 ml per minute to the mass spectrometer. The mass spectrometer was operated in the multiple reaction-monitoring mode using nitrogen as the collision gas and a collision energy of 20 eV. The transitions from *m*/*z* 284.2 (M^+^ H^+^) to 168.1 for 8-oxodG and 268.2 (M^+^ H^+^) to 152.1 for dG were monitored with a residence time of 350 ms. The samples were quantified by comparing the peak areas of the standards to the peak areas of the samples. Using a 50-µl injection volume, the limit of detection using this method is typically 1 µg/l for 8-oxodG and 50 µg/l for dG. Some interference from other components present in the sample has been noted, particularly in the dG determination.

### 3.10 Statistical analysis

All statistical calculations were performed and the graphs were plotted using GraphPad Prism software version 6.0. In the graphs that chart the 8-oxodG ratios, all of the points cannot be shown because some of the 8-oxodG/dG ratios deviated from the others at the bottom of the graphs. Therefore, according to the statistical guide of GraphPad Prism software version 6.0, the 8-oxodG/dG ratios were first transformed to logarithms. The logarithms of the 8-oxodG/dG ratios of the various groups of patients were evaluated for the normality of the distribution by the D'Agostino & Pearson omnibus normality test to decide whether a nonparametric rank-based analysis or a parametric analysis should be used. Statistically significant differences in the unpaired hepatic 8-oxodG/dG ratios of the two groups of patients were compared with the Mann–Whitney *U* test for data without a normal distribution or with the unpaired *t* test for data with a normal distribution. Statistically significant differences between the logarithms of paired hepatic 8-oxodG/dG ratios of non-malignant liver tissues and those of malignant tissues from the same HCC patients were compared with the Wilcoxon matched-pairs signed rank test for the data without a normal distribution or with the paired *t* test for the data with a normal distribution. *P* values less than 0.05 were considered statistically significant. The box-whisker plots expressed the logarithms of the 8-oxodG/dG ratios. The results were expressed as the means±standard derivation or as medians.

## 4 Results

### 4.1 DNA extraction

In this study, to determine the effects of temperature on the formation of 8-oxodG during DNA extraction, human liver DNA was extracted using the cold 4 M GTC method (4 °C) and the warm (37 °C) RNase A/proteinase K method. These DNA samples together with commercial calf DNA used as a control (maintained at 25 °C in air) were hydrolyzed and the 8-oxodG levels were measured by HPLC-MS/MS. The results indicated that the cold 4 M GTC method had the lowest ratios of 8-oxodG/dG, while the ratio from the same liver tissue using the warm RNase A/proteinase K method was increased by approximately two-fold and the 8-oxodG/dG ratios from the commercial calf DNA exposed to room air were 31-fold higher (see Table 2). Additionally, the DNA yields of the eight samples were significantly different; the largest DNA yield was 7.57 µg/mg, and the smallest DNA yield was 0.19 µg/mg. The 8-oxodG levels in 2 samples could not be detected by HPLC-MS/MS, although Table 2 showed that sufficient amounts of DNA were used.

### 4.2 DNA hydrolysis

One-hundred micrograms of commercial calf thymus DNA was hydrolyzed with 1 µg, 5 µg, 10 µg or 20 µg of nuclease P1 and 1 unit of alkaline phosphatase at 5 different temperatures and incubation conditions. The results from these experiments are shown in Table 3. These data revealed that the dG yields in Conditions 1, 2 and 3 were higher than those in Conditions 4 and 5. Second, the DNA was hydrolyzed at similar levels with all concentrations of nuclease P1 (1 to 20 µg) in these three conditions. Third, the 8-oxodG/dG ratios in Conditions 1 and 3 were less than those in Conditions 2 and 5. This indicated that Conditions 1 and 3 resulted in reduced 8-oxodG production compared to Conditions 2 and 5. A previous study supported the use of Condition 1 to hydrolyze the DNA for 8-oxodG measurements [48], and a decision was made to hydrolyze the DNA using Condition 1, in which the DNA was incubated with 2 µg of nuclease P1 and 1 unit of alkaline phosphatase for 1 h at 50 °C.

### 4.3 Comparisons of the logarithms of the 8-oxodG/dG ratios in the non-malignant liver tissues to those in the malignant tissues of HCC patients

**Fig. 1:**
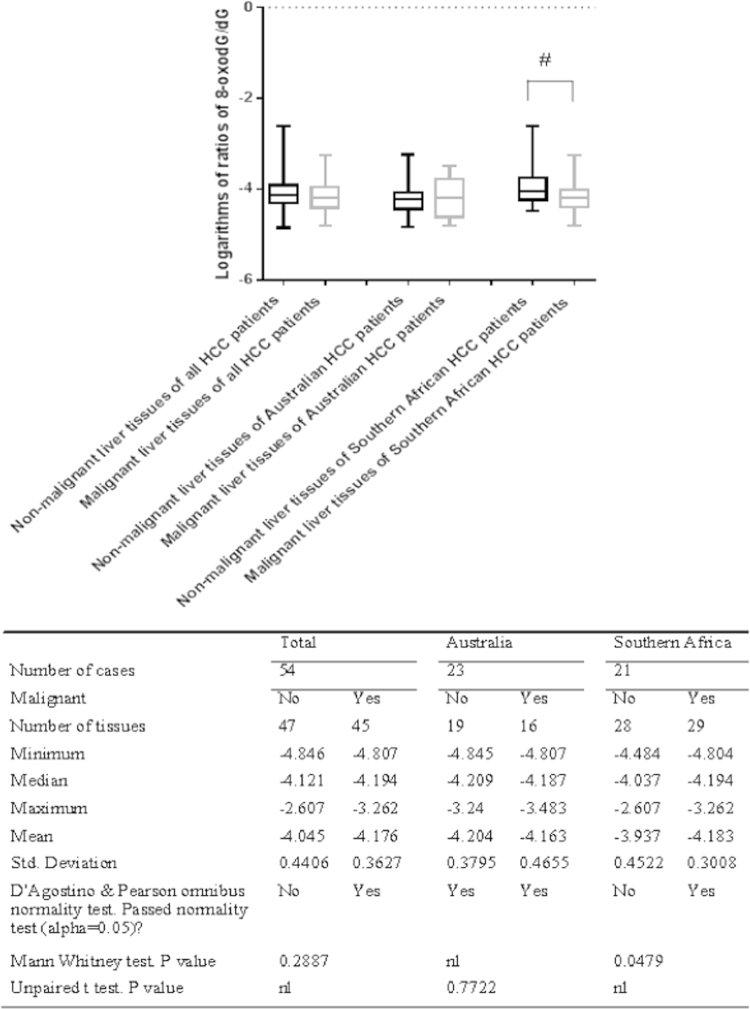
Comparisons of the logarithms of the 8-oxodG/dG ratios in non-malignant liver tissues versus those in malignant tissues of HCC patients. # indicates that the 8-oxodG/dG ratios in the non-malignant liver tissues were significantly increased compared to those in the malignant tissues of Southern African HCC patients.

Among the total 54 HCC cases, both non-malignant liver tissues and malignant tissues are available for some cases, while only non-malignant liver tissues or malignant tissues are available for other cases. Each tissue was used to measure the 8-oxodG and dG levels 1–6 times, and then, the 8-oxodG/dG ratios in each tissue were averaged. The 8-oxodG/dG ratios were first transformed to logarithms. The distribution of the logarithms of the 8-oxodG/dG ratios in the malignant and non-malignant liver tissues was analyzed for normality with the D'Agostino and Pearson omnibus normality test. Only the distributions of the logarithms of the 8-oxodG/dG ratios in the non-malignant tissues and malignant tissues from Australian HCC patients were normal, while those of the total HCC cases or the Southern African HCC patients were not (see Fig. 1). Therefore, statistically significant differences between the logarithms of the 8-oxodG/dG ratios in the non-malignant liver tissues and those in the malignant tissues of Australian HCC patients were evaluated with the unpaired *t* test, while those of the total cases and the Southern African HCC patients were tested with the Mann–Whitney *U* test.

The statistical analysis showed that there were significant differences between the logarithms of the 8-oxodG/dG ratios in the non-malignant tissues and those in the malignant tissues of Southern African HCC patients (*p*=0.0479), while there were no significant differences in the total cases (*p*=0.2887) or Australian HCC patients (*p*=0.7722) (see Fig. 1).

### 4.4 Paired comparisons of the logarithms of the 8-oxodG/dG ratios between non-malignant liver tissues and malignant tissues of the same HCC patients

**Fig. 2:**
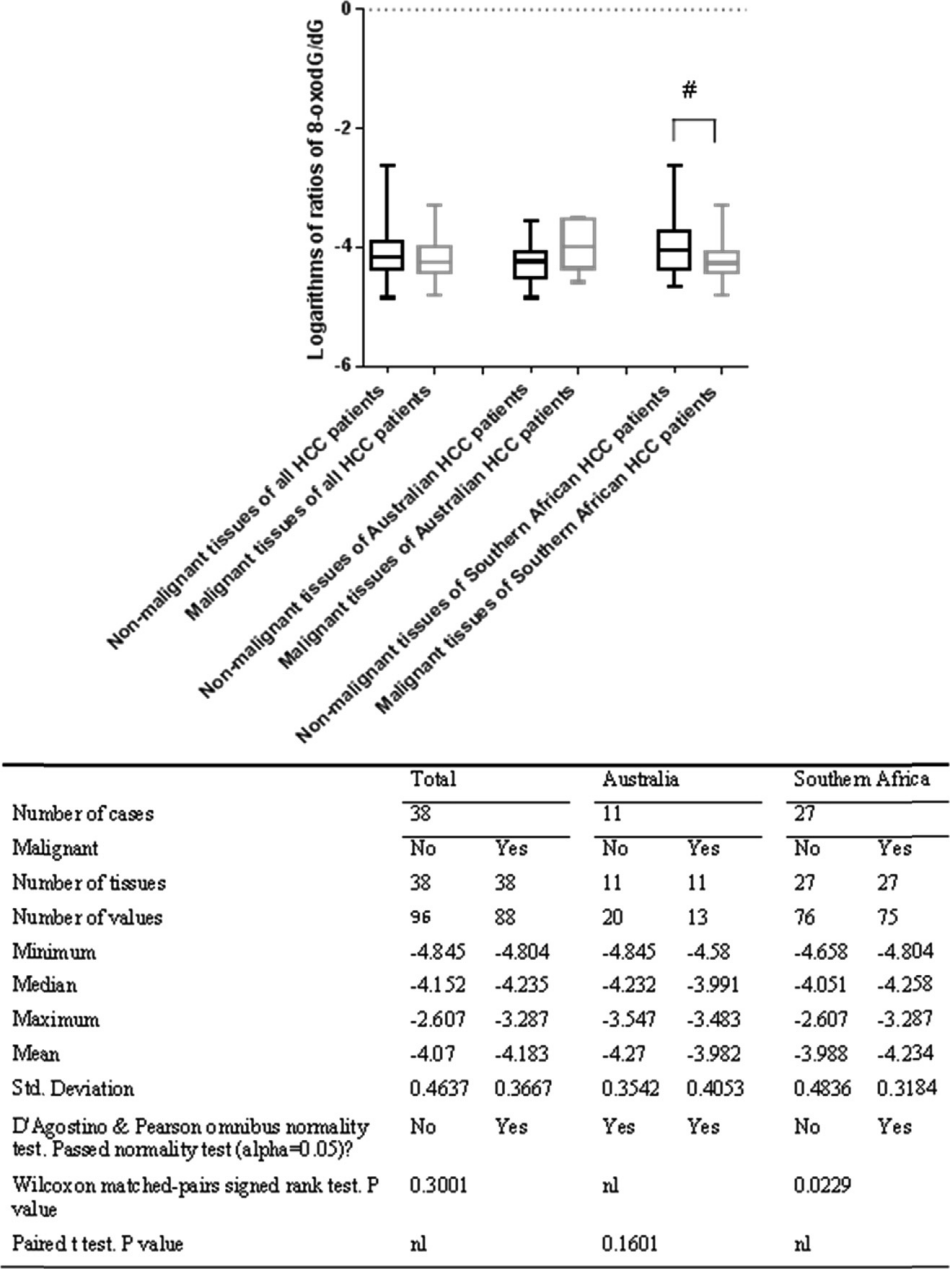
Paired comparisons of the logarithms of the 8-oxodG/dG ratios in non-malignant liver tissues to those in malignant tissues of the same HCC patients. # indicates that the 8-oxodG/dG ratios in the non-malignant liver tissues were significantly increased compared to those in the malignant tissues of Southern African HCC patients.

In the previous sections, the logarithms of mixtures of the 8-oxodG/dG ratios in all non-malignant liver tissues were compared to those of all malignant tissues. In this section, the logarithms of the 8-oxodG/dG ratios in non-malignant liver tissues were compared to those in malignant tissues from the same HCC patients. The distributions of the logarithms of the 8-oxodG/dG ratios in non-malignant and malignant liver tissues from the same HCC patients were analyzed for normality with the D'Agostino & Pearson omnibus normality test. The results showed that the distributions of the logarithms of the 8-oxodG/dG ratios in non-malignant liver tissues and malignant tissues of Australian HCC patients were normal, while those in the total HCC patients or Southern African HCC patients were not normal (see Fig. 2). Therefore, the statistically significant differences between the logarithms of the 8-oxodG/dG ratios in non-malignant liver tissues and those in malignant tissues of Australian HCC patients were compared using the paired *t* test, while those in the total HCC patients and Southern African HCC patients were compared using the Wilcoxon matched-pairs signed rank test.

The statistical analysis showed that there was a significant increase in this ratio in the non-malignant liver tissue of Southern African HCC patients (*p*=0.0229), while there was no significant difference in the total cases (*p*=0.3001) or Australian patients (*p*=0.1601) (see Fig. 2)

### 4.5 Comparison of the logarithms of the 8-oxodG/dG ratios in non-cirrhotic and cirrhotic human liver tissue from HCC patients

**Fig. 3:**
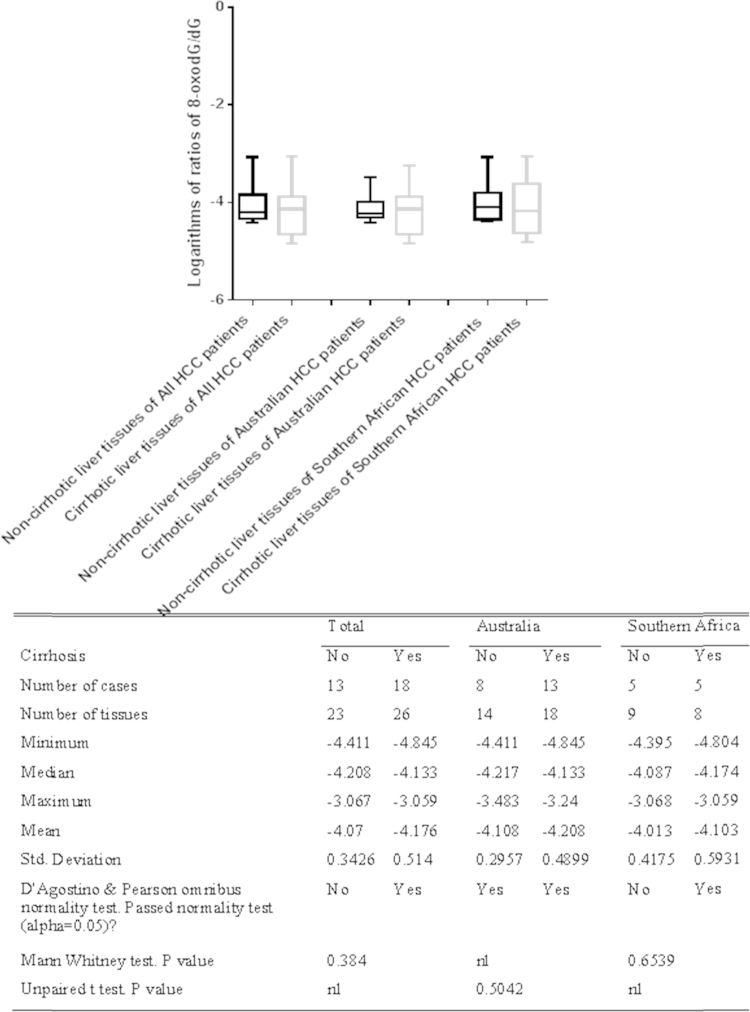
Comparison of the logarithms of the 8-oxodG/dG ratios in non-cirrhotic liver tissues to those in cirrhotic liver tissues of 31 HCC patients.

When the HCC patients were separated according to the presence of cirrhosis, the normalities of the logarithms of the 8-oxodG/dG ratios in the non-cirrhotic liver tissues and cirrhotic liver tissues of 31 HCC patients were evaluated with the D’Agostino and Pearson omnibus normality test. The results showed that the distributions of the logarithms of the 8-oxodG/dG ratios in non-cirrhotic liver tissues the cirrhotic liver tissues of Australian HCC patients were normal, but those of the total HCC cases or Southern African HCC patients were not normal. Therefore, the statistically significant differences between the non-cirrhotic and cirrhotic liver tissues of Australian HCC patients were tested with the unpaired *t* test, while the differences in the total HCC patients and Southern African HCC patients were compared with the Mann–Whitney *U* test. The results demonstrated that there was no significant difference between the 8-oxodG/dG ratios in the cirrhotic and non-cirrhotic liver tissues from the total HCC patients (*p*=0.384), Australian HCC patients (*p*=0.5042) or Southern African HCC patients (*p*=0.6539) (see Fig. 3).

### 4.6 Comparison of the logarithms of the 8-oxodG/dG ratios based the on underlying cause of liver disease

**Fig. 4:**
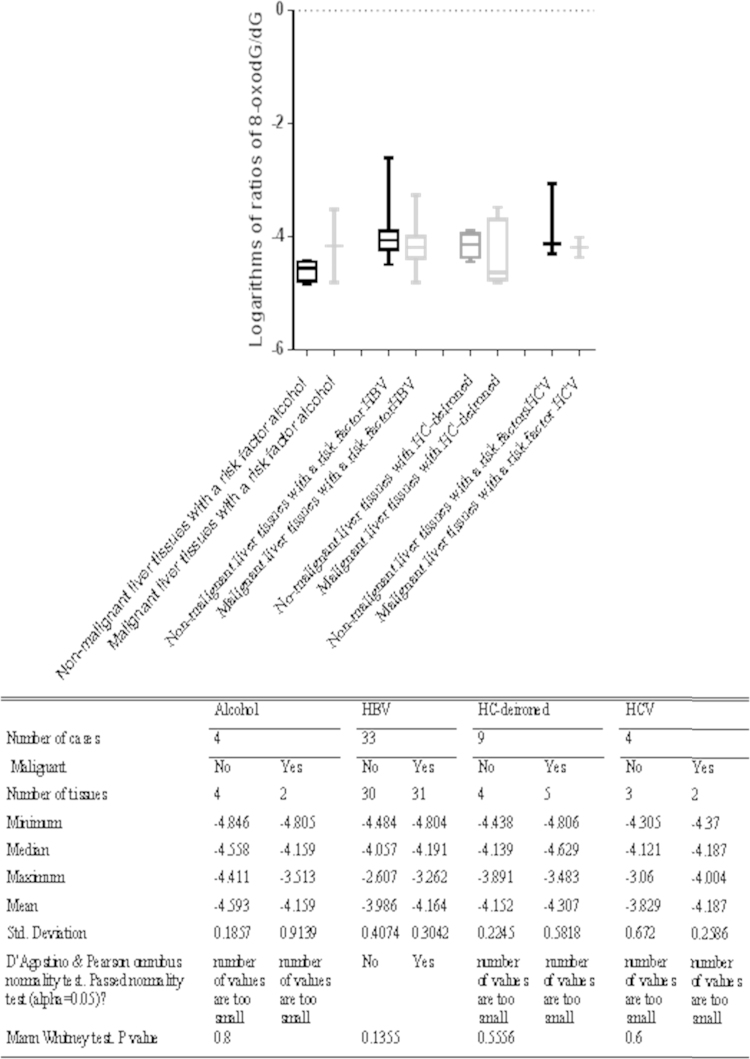
Comparison of the logarithms of the 8-oxodG/dG ratios in non-malignant liver tissues to those in malignant tissues separated by the underlying cause of liver disease.

The logarithms of the 8-oxodG/dG ratios in patient groups classified according to their underlying liver disease were not normally distributed. The logarithms of the 8-oxodG/dG ratios in malignant and non-malignant liver tissue in various chronic liver diseases were compared with the Mann–Whitney *U* test. This analysis revealed that there were no significant differences between the logarithms of the 8-oxodG/dG ratios in non-malignant liver tissues and those in malignant tissues in these liver diseases (see Fig. 4).

## 5 Discussion

### 5.1 8-oxodG/dG exhibited an increasing trend in non-malignant liver tissues compared to malignant liver tissues

In this study, there was a trend towards increased 8-oxodG/dG ratios in non-malignant liver tissues compared to malignant liver tissues, although the difference was not significant. This trend was most obvious in Southern African patients (see Figs. 1 and 2). Nevertheless, the data do not support the hypothesis that the hepatic 8-oxodG/dG ratios are increased in non-malignant liver tissues compared to HCC. The 8-oxodG/dG ratios found in this study had skewed distributions for most of the analyzed groups, with most groups having clustered 8-oxodG/dG values, and a small number of outlier samples were widely separated from the group.

At least one previous study has observed that the 8-oxodG levels markedly increase in non-malignant liver tissue compared to the tumors in patients with HCC. For example, the difference between malignant liver tissue and tumors was observed in both American and Southern African patients with HCC [44]. Another study has shown that the 8-oxodG levels in non-malignant liver tissues are significantly increased compared to the corresponding HCC tissues of the same subject and that the 8-oxodG levels are significantly increased in non-malignant tissues with moderate inflammation compared to those with mild or no inflammation [45]. A positive correlation between the 8-oxodG concentration in non-malignant liver tissue and serum alanine aminotransferase activity has also been observed [46]. However, several previous studies have revealed that the 8-oxodG levels are decreased in non-malignant livers compared to the malignant liver tissues from the same patient. For example, 8-oxodG concentrations have been reported to be decreased in the cancer-free surrounding tissues compared to the malignant lung tissues [47].

A number of studies have shown that 8-oxodG is the main ROS- and RNS-oxidized form of DNA base; moreover, 8-oxodG is a pre-mutagenic agent, and chronic liver inflammation leads to the production of ROS and RNS [48], [49]. This means that the presence of increased 8-oxodG levels in liver tissues adjacent to HCC implicates 8-oxodG as a link between chronic hepatic inflammation and hepatic carcinogenesis; the oxidative DNA damage in chronic hepatic inflammation may lead to the malignant transformation of hepatocytes. However, this study identified an increasing trend in the 8-oxodG/dG ratio in non-malignant liver tissues compared to malignant tissues of HCC patients (Figs. 1 and 2), particularly in HCC patients with HBV (Fig. 4), but these trends were not very significant. This study also revealed that there was no significant difference in the 8-oxodG/dG ratios between the cirrhotic and non-cirrhotic liver tissue of patients with HCC (Fig. 3) or among HCC patients with various risk factors, including HBV, HCV, alcohol, Allagile's syndrome, and haemochromatosis (both before and after de-ironing) (Fig. 4). Moreover, most comparisons of the 8-oxodG/dG ratios showed that there were no significant differences between the patient groups and tissue types.

Previous reports show that the 8-oxodG/dG ratios in non-malignant liver tissues and malignant HCC tumors were affected by the formation, oxidation and deletion of 8-oxodG in inflammatory tissues. In liver inflammation, the activated immune system generates excessive ROS and RNS, which can not only form 8-oxodG but can also decompose 8-oxodG. Recent studies have shown that 8-oxodG easily reacts with ONOO^−^ [50], ^1^O_2_ [51], [52] and Fe^2+^ [53] to form secondary oxidative products, as it possesses a lower redox potential than guanosine [54]. For example, 8-oxodG is oxidized by ONOO^−^, at least 1000 times faster than G [55]. DNA repair enzymes can also remove 8-oxodG [56].

Recent reports also revealed that 8-oxodG is not a single pro-mutagenic agent in hepatocarcinogenesis. To date, more than 100 DNA lesions in addition to 8-oxodG have been identified [57], [58], and other various oxidative products of bases and secondary oxidative products of 8-oxodG are also pro-mutagenic agents [55]. Some oxidative products of bases have higher mutation frequencies. For example, the frequencies of the G to T conversion for oxazolone and spiroiminodihydantoin are far higher than those of 8-oxodG [59], [60].

Based on above observations, the 8-oxodG/dG ratio did not reflect a correlation between oxidative DNA damage in chronic liver inflammation and hepatocarcinogenesis.

### 5.2 Many factors affected the examinations of the statistically significant differences

In this study, there was no statistically significant difference in the 8-oxodG/ dG ratios between non-malignant and malignant liver tissue from Australian patients or between cirrhotic and non-cirrhotic liver tissues of patients with HCC (see Fig. 3). The 8-oxodG/dG ratios in non-malignant liver tissues of HCC patients with HBV were significantly increased compared to the tumors in these patients, but significant differences were not found in HCC patients with other chronic diseases, including alcohol, Allagile's syndrome, and haemochromatosis (both before and after de-ironing) (see Fig. 4). Most comparisons of the 8-oxodG/dG ratios showed that there were no significant differences between the patient groups and tissue types. There are may be several reasons for these observations. One possible reason was that we used an insufficient number of tissues and cases, which may have affected these comparisons. The tissues from the Australian patients were extremely valuable and in limited supply. Additionally, there were only 4 cases of HCC patients with HCV, 4 cases of HCC patients with alcohol, and 1 case of an HCC patient with Allagile's syndrome. This, together with the limited availability of sample material in some groups, made comparisons of the 8-oxodG/dG ratios difficult. The second reason is that there is no correlation between the malignant grades and these risk factors. For example, one study found that there was no positive correlation between the hepatic iron content and 8-oxodG concentration [45]. The hepatic Fe and hepatic 8-oxodG levels are not correlated [44]. However, other studies have shown a link between alcohol, iron and 8-oxodG. The third reason is that there were variations in the degree of inflammation/liver injury; moreover, the presence of parenchymal elements, such as connective tissue and blood vessels, in different tissue samples may have impacted the final DNA yield and the 8-oxodG/dG ratio.

Previous studies have indicated that the production of artefactual 8-oxodG caused by high temperature, incomplete hydrolysis of DNA and imprecise 8-oxodG measurements lead to a lack of significant differences in the 8-oxodG/dG ratios between non-malignant liver tissues and tumoural tissues. This study attempted to remove these confounds by optimizing the conditions for DNA isolation, DNA hydrolysis and the 8-oxodG measurements.

### 5.3 Inadequate DNA extraction led to an inaccurate DNA yield

During the optimization of the DNA extraction procedure, there were problems in the earliest experiments, with large variations in the DNA yield between samples, and there was a failure to identify 8-oxodG by HPLC. This was because the tissues were not completely homogenized or the DNA was lost during the procedures. Thereafter, special care was taken so that the tissues were homogenized gently to avoid rupturing the nuclear membrane, which resulted in the loss of the DNA during the nuclei precipitation step. The homogenization tube was washed with a small amount of homogenization buffer two or three times and the residual homogenized solution was collected into the sample. If large amounts of lipid were present during centrifugation, the centrifugation force was increased to pellet the nuclei. The crude nuclei pellets were dissolved in cold 4 M GTC, which suppressed any of the DNase enzymes present in the solution that otherwise would have hydrolyzed the DNA [61].

The nuclei pellets were purified with Sevag solution. Sevag solution consisted of 1 volume of isoamyl alcohol and 24 volumes of chloroform. The isoamyl alcohol reduced foaming, aided the separation, and maintained the stability of the layers of the centrifuged, deproteinized solution. Chloroform causes surface denaturation of proteins. It is worth noting that chloroform could react with the microcentrifuge tubes, resulting in sample leakage during centrifugation. To avoid this problem, the DNA solution was immediately transferred to a fresh tube after centrifugation.

During DNA extraction, PLG tubes were used to separate the organic phase containing proteins and the aqueous phases containing DNA. One difference in the PLG tubes compared to normal centrifuge tubes is that these tubes contain a phase block gel that can separate aqueous and organic media based on their density differences. After centrifugation, the denatured protein and organic solutions are effectively trapped in the lower organic phases of the PLG by the gel, while the DNA remains in upper aqueous phases and can be easily removed with a pipette. The use of the PLG tubes resulted in a DNA-containing phase that could be easily pipetted, resulting in the recovery of 20 to 30% more nucleic acid than with traditional methods. After cold isopropanol was added to the solution, DNA rapidly precipitated out of solution as a stringy gelatinous clump, unless it was sheared. If the DNA was sheared, it was precipitated by placing the tube in a −20 °C freezer from 20 min to overnight.

Table 2 showed that the DNA yields exhibited large variations among samples, in which the highest DNA yield was 7.57 µg/mg, whereas the smallest DNA yield was 0.19 µg/mg. Obviously, these data did not represent the actual DNA concentrations. This was because the DNA precipitate was incompletely dissolved. Large DNA aggregates remaining in the tube produced abnormally high or low UV absorbance readings, leading to an erroneous DNA concentration. To avoid this problem, the DNA precipitate was carefully dissolved by pipetting it repeatedly to obtain a homogeneous preparation.

Additionally, the 8-oxodG levels in 2 samples could not be detected by HPLC-MS/MS (see Table 2). One reason could be that insufficient DNA concentrations were used, although the data showed that their DNA concentrations were very high. As mentioned in a previous report, if 1 mg of human liver tissues yields approximately 1 µg of DNA [32], 50 mg of tissue would yield 10 fmol of 8-oxodG, which is close to the detection limit of 7.5 fmol for HPLC-MS/MS. Therefore, if the tissues were not completely homogenized or DNA was lost during an extraction, 8-oxodG could not be detected by HPLC-MS/MS. Therefore, great care was taken to ensure that as much tissue was available as possible.

### 5.4 High temperature increased artefactual 8-oxodG production during DNA extraction

In this study, to determine the effects of temperature on the formation of 8-oxodG during DNA extraction and hydrolysis, human liver DNA was extracted with the cold M GTC method (4 °C) and a warm (37 °C) RNase A/proteinase K method. These DNA samples, together with commercial calf DNA (maintained at 25 °C and exposed oxygen in air for a long time) as a control, were hydrolyzed and the 8-oxodG levels were measured by HPLC-MS/MS. The results indicated that the cold 4 M GTC method produced the lowest 8-oxodG yield, while the levels from the same liver tissue using the warm RNase A/proteinase K method were increased approximately two-fold, and the 8-oxodG/dG ratios from the commercial calf DNA exposed to room air were increased 35-fold (see Table 2). These results indicated that the low temperature DNA extraction method reduced the formation of 8-oxodG during the procedure. In addition, the much higher 8-oxodG/dG values in commercial calf DNA compared to those of the DNA that had been freshly extracted using both the cold M GTC method and the warm RNase A/proteinase K may have resulted from the exposure of the DNA to the oxygen in air during the longer incubation at room temperature, stressing the need for refrigeration of the extracted DNA and minimizing its exposure to air prior to analysis. Therefore, the cold 4 M GTC method was used to extract the DNA from the human liver tissues in this study. Additionally, samples were stored in a −80 °C freezer, and the tissues were dissected and homogenized and DNA was extracted in a cold room. All other procedures were performed in a cold room as much as possible.

### 5.5 High temperature increased artefactual 8-oxodG production, whereas low temperature and a short incubation time incompletely hydrolyzed the DNA

The dG yields from 100 µg of commercial calf thymus DNA that was hydrolyzed with 1 µg, 5 µg, 10 µg or 20 µg of nuclease P1 and 1 unit of alkaline phosphatase under Conditions 1, 2 and 3 were more similar and larger than those under Conditions 4 and 5 (see Table 3). This indicated that the dG concentrations were not correlated to those of nuclease P1 under Conditions 1, 2 and 3. This observation was consistent with a previous study in which 10 to 25 µg of nuclease P1 did not increase DNA hydrolysis [32]. Furthermore, 1 µg of nuclease P1 completely hydrolyzed 100 µg of commercial calf thymus DNA under Conditions 1, 2 and 3.

Conditions 4 and 5 produced less dG and 8-oxodG compared to Conditions 1, 2 and 3, but the 8-oxodG/dG ratios were still high. This indicated that incomplete DNA hydrolysis led to an overestimation of the 8-oxodG levels. Condition 4 produced incomplete hydrolysis due to the short hydrolysis time, in which nuclease P1 hydrolyzed DNA for only 10 min, whereas the time increased to 1 h under Conditions 1, 2 and 3 and 0.5 h under Condition 5. The incomplete DNA hydrolysis under Condition 5 was due to the low temperature. A previous study has also revealed that DNA hydrolysis is poor below 37 °C [32]. Condition 2 produced more 8-oxodG than Conditions 1, 3, 4 and 5. This indicated that high temperature produced 8-oxodG.

In summary, 1 µg of nuclease P1 completely hydrolyzed 100 µg of DNA in 1 h at 50 °C. However, this hydrolysis was not complete with a short incubation time (≤10 min) even at 65 °C. An additional concern with heating the samples to 100 °C during hydrolysis was that this appeared to lead to the formation of artefactual 8-oxodG. Conditions 1 and 3 produced similar amounts of dG and 8-oxodG, but there was an additional hour-long incubation at 37 °C for hydrolysis by alkaline phosphatase. The longer hydrolysis time and room temperature increased the risk of forming artefactual 8-oxodG. For these reasons, the DNA of the HCC patients in this study was hydrolyzed with 1 unit of alkaline phosphatase and 1 µg of nuclease P1 for one hour at 50 °C.

### 5.6 Limitations of this study

The experiments were limited to approximately 50 mg of liver tissue per sample. This was necessary because of the scarcity of these tissues. This amount of tissue gave a yield of approximate 10 fmol of 8-oxodG, which was close to the detection limit of 7.5 fmol for HPLC-MS/MS. If tissues were not completely homogenized, DNA was lost during an extraction step, or DNA was incompletely hydrolyzed, 8-oxodG could not be detected by HPLC-MS/MS. Therefore, great care was taken to ensure that as much tissue was available as possible. Second, the temperature at which the samples were processed required careful control because high temperatures produced artefactual 8-oxodG values. In this experiment, the samples were stored in a −80 °C freezer, and the tissues were dissected and homogenized and DNA was extracted in a cold room. All other procedures were performed in a cold room, as much as possible; however, the DNA was hydrolyzed at 50 °C and the 8-oxodG levels were measured by HPLC-MS/MS at room temperature. These temperatures could potentially produce artefactual 8-oxodG. It is not known how much 8-oxodG was produced during the experimental procedures and whether this influenced our results. Third, it is not possible to determine whether the DNA was completely hydrolyzed with nuclease P1 and alkaline phosphatase, even if all of the procedures are implemented to allow complete DNA hydrolysis. If the DNA is not completely hydrolyzed, the ratios of 8-oxodG/dG will be altered.

These temperatures could potentially produce artefactual 8-oxodG. It is not known how much 8-oxodG was produced during the experimental procedures and whether this influenced our results. Third, it is not possible to determine whether the DNA was completely hydrolyzed with nuclease P1 and alkaline phosphatase, even if all of the procedures are implemented to allow complete DNA hydrolysis. If the DNA is not completely hydrolyzed, the ratios of 8-oxodG/dG will be altered. Therefore, compared to previous studies, this experiment was performed very carefully to reflect the actual 8-oxodG values.

## 6 Summary

This study revealed that the 8-oxodG/dG ratios tended to be higher in most non-malignant liver tissues than those in HCC tissues, although this was not statistically significant. It also appeared that the ratio was higher in the non-malignant liver tissue from Southern African patients, but there was no difference in the 8-oxodG/dG ratios between non-malignant liver tissues and tumors of Australian HCC patients. Additionally, this study also revealed an increasing trend for 8-oxodG/dG ratios in non-malignant liver tissues compared to tumoural tissues of patients with HBV. These findings confirmed that there was an association between oxidative DNA damage and chronic liver inflammation, but there was no dose-dependent relationship between the 8-oxodG levels and hepatocarcinogenesis. Due to the limitations of this study, significant differences in the 8-oxodG/dG ratios between the non-cirrhotic and cirrhotic non-malignant liver tissues were not observed.

The methods used in these experiments were suitable for measuring the 8-oxodG levels from human liver tissues. The cold 4 M GTC method produced a sufficient amount of DNA from 50 mg of the tissues used for the analysis. Fifty micrograms of DNA were sufficiently hydrolyzed using 1 µg of nuclease P1 and 1 unit of alkaline phosphatase. The levels of artefactual 8-oxodG produced using the optimized methods in this study were demonstrated to be lower than those produced using the methods described in previous studies. Despite these limitations, this study more thoroughly controlled for artefactual 8-oxodG/dG production and more accurately reflects the actual 8-oxodG/dG levels in the samples than previous studies.

## Figures and Tables

**Table 1:** Characteristics of the patients with hepatocellular carcinoma.

	Cases	Available tissue		Cirrhosis			Risk factors for chronic liver disease*					
	Total	Non-malignant liver tissues	Tumor	Non-cirrhosis	Cirrhosis	UK^a^	HBV	HC	HCV	Alcohol	Allagile's syndrome	Nil
Total	54	47	45	13	18	23	33	9	4	4	1	3
Australia	23	19	16	8	13	2	3	9	3	4	1	3
Southern Africa	31	28	29	5	5	21	30	–	1	–	–	–

a UK – Unknown – no clinical data available.

* HBV – Hepatitis B; HC – Haemochromatosis; HCV – Hepatitis C; Nil – No identified cause of chronic liver disease.

**Table 2:** DNA extraction with the warm proteinase K digestion and cold 4 M GTC methods.

Method	Tissues	Tissue amount (mg)	DNA amount (µg)	Yields of DNA (µg/mg)	dG (µg/l)	8-oxodG (µg/l)	8-oxodG/dG (10^−5^)
The warm proteinase K digestion method	Normal A	93	398	4.28	13,200	0.6	4.5
Normal B	50	269.2	5.38	263	n/a^a^	n/a^a^
Tumor 1	70	313	4.47	9620	3	3.1
Tumor 2	60	280	4.67	14,830	0.7	4.7
The cold 4 M GTC method	Normal A	63	477	7.57	18,000	0.4	2.2
Normal B	55	279	5.07	4620	n/a	n/a
Tumor 1	125	n/a^a^	n/a	12,500	0.2	1.6
Tumor 2	60	11.9	0.19	12,150	0.4	3.3
Positive control	Calf thymus DNA	–	100		9800	7.6	77.5
Calf thymus DNA	–	60		8250	5.9	71.5

a not detected

**Table 3:** Enzymatic hydrolysis of calf Commercial DNA under 5 different conditions.

Experimental conditions	Nuclease P1 (µg)	dG (mg/l)	8-oxodG (µg/l)	8-oxodG/dG (10^−5^)
1, nuclease P1+alkaline phosphatase for 1 h at 50 °C	1	44.5	35.2	79
5	44.9	40.8	90
10	41.2	33.6	81
20	39.3	33.6	85
2, heated for 5 min at 100 °C, nuclease P1+alkaline phosphatase for 1 h at 50 °C	1	46.3	49.3	106
5	46.5	47.3	101
10	4.8	2.1	43
20	43.1	43.7	101
3, nuclease P1 for 1 h at 50 °C, alkaline phosphatase for 1 h at 37 °C	1	40.1	29.4	73
5	41.8	32.2	77
10	39.8	31.7	79
20	41.8	33.1	79
4, nuclease P1 for 10 min at 65 °C, alkaline phosphatase for 1 h at 37 °C	1	38.2	28.3	74
5	42.5	36.6	85
10	29.5	25.9	87
20	28.1	22.4	79
5, nuclease P1 for 0.5 h at 37 °C, alkaline phosphatase for 1 h at 37 °C	1	29.5	23.1	78
5	38.1	49	128
10	31.4	33	105
20	31.3	32.6	104

## References

[1] Kojiro M., Wang X.W., Grisham J.W., Thorgeirsson S.S. (2011). Pathology of hepatocellular carcinoma. Molecular Genetics of Liver Neoplasia.

[2] Cornellà H., Alsinet C., Villanueva A. (2011). Molecular Pathogenesis of Hepatocellular Carcinoma. Alcohol.: Clin. Exp. Res..

[3] Jain S. (2010). Molecular genetics of hepatocellular neoplasia. Am. J. Transl. Res..

[4] Ziech D. (2011). Reactive Oxygen Species (ROS)--Induced genetic and epigenetic alterations in human carcinogenesis. Mutat. Res./Fundam. Mol. Mech. Mutagen..

[5] DeMott M.S., Dedon P.C., Geacintov N.E., Broyde S. (2010). Chemistry of inflammation and DNA damage: biological impact of reactive nitrogen species. The Chemical Biology of DNA Damage.

[6] Vogelstein B., Kinzler K.W. (2004). Cancer genes and the pathways they control. Nat. Med..

[7] Lopez-Lazaro M. (2010). A new view of carcinogenesis and an alternative approach to cancer therapy. Mol. Med..

[8] Laurent-Puig P., Zucman-Rossi J. (2006). Genetics of hepatocellular tumors. Oncogene.

[9] Thorgeirsson S.S., Grisham J.W. (2002). Molecular pathogenesis of human hepatocellular carcinoma. Nat. Genet..

[10] Kitada T. (2001). In situ detection of oxidative DNA damage, 8-hydroxydeoxyguanosine, in chronic human liver disease. J. Hepatol..

[11] Kato J. (2001). Normalization of elevated hepatic 8-Hydroxy−2’-deoxyguanosine levels in chronic hepatitis C patients by phlebotomy and low iron diet. Cancer Res..

[12] Coussens L.M., Werb Z. (2002). Inflammation and cancer. Nature.

[13] Bolondi L., Gramantieri L. (2011). From liver cirrhosis to HCC. Intern. Emerg. Med..

[14] Llovet J.M., Burroughs A., Bruix J. (2003). Hepatocellular carcinoma. Lancet.

[15] Monga S.P.S., Cubero F.J., Trautwein C. (2011). Oxidative Stress and Liver Injury. Molecular Pathology of Liver Diseases.

[16] Federico A. (2007). Chronic inflammation and oxidative stress in human carcinogenesis. Int. J. Cancer.

[17] Pârvu A.E. (2005). Nitric oxide in patients with chronic liver diseases. Romanian J. Gastroenterol..

[18] Bonavida B., Teicher B.A. (2010). Prognostic Significance of iNOS in Hepatocellular Carcinoma, in Nitric Oxide (NO) and Cancer.

[19] Dizdaroglu M., Jaruga P. (2012). Mechanisms of free radical-induced damage to DNA. Free Radic. Res..

[20] Lonkar P., Dedon P.C. (2011). Reactive species and DNA damage in chronic inflammation: reconciling chemical mechanisms and biological fates. Int. J. Cancer.

[21] Dedon P.C., Tannenbaum S.R. (2004). Reactive nitrogen species in the chemical biology of inflammation. Arch. Biochem. Biophys..

[22] Kryston T.B. (2011). Role of oxidative stress and DNA damage in human carcinogenesis. Mutat. Res./Fundam. Mol. Mech. Mutagen..

[23] Shibutani S., Takeshita M., Grollman A.P. (1991). Insertion of specific bases during DNA synthesis past the oxidation-damaged base 8-oxodG. Nature.

[24] Duarte V., Muller J.G., Burrows C.J. (1999). Insertion of dGMP and dAMP during in vitro DNA synthesis opposite an oxidized form of 7,8-dihydro−8-oxoguanine. Nucl. Acids Res..

[25] Kawanishi S. (2006). Oxidative and nitrative DNA damage in animals and patients with inflammatory diseases in relation to inflammationrelated carcinogenesis. Biol. Chem..

[26] Gedik C.M. (2002). Oxidative stress in humans: validation of biomarkers of DNA damage. Carcinogenesis.

[27] Escodd C., Gedik, Collins A. (2004). Establishing the background level of base oxidation in human lymphocyte DNA: results of an interlaboratory validation study. FASEB J..

[28] Sova H. (2010). 8-Hydroxydeoxyguanosine: a new potential independent prognostic factor in breast cancer. Br. J. Cancer.

[29] Shen J. (2007). 8-Hydroxy−2′-deoxyguanosine (8-OH-dG) as a potential survival biomarker in patients with nonsmall-cell lung cancer. Cancer.

[30] Karihtala P. (2009). DNA Adduct 8-Hydroxydeoxyguanosine, a Novel Putative Marker of Prognostic Significance in Ovarian Carcinoma. Int. J. Gynecol. Cancer.

[31] Olinski R. (2003). Oxidative DNA damage in cancer patients: a cause or a consequence of the disease development?. Mutat. Rese./Fundam. Mol. Mech. Mutagen..

[32] Hofer T., Moller L. (2002). Optimization of the workup procedure for the analysis of 8-Oxo−7,8-dihydro−2′-deoxyguanosine with electrochemical detection. Chem. Res. Toxicol..

[33] Evans M.D. (2010). Toward consensus in the analysis of urinary 8-oxo−7,8-dihydro−2’-deoxyguanosine as a noninvasive biomarker of oxidative stress. FASEB J..

[34] Hofer T., Moller L. (1998). Reduction of Oxidation during the Preparation of DNA and Analysis of 8-Hydroxy−2’-deoxyguanosine. Chem. Res. Toxicol..

[35] Gedik C.M., Wood S.G., Collins A.R. (1998). Measuring oxidative damage to DNA; HPLC and the comet assay compared. Free Radical Research.

[36] Weimann A., Belling D., Poulsen H.E. (2001). Measurement of 8-oxo−2’-deoxyguanosine and 8-oxo−2’-deoxyadenosine in DNA and human urine by high performance liquid chromatography-electrospray tandem mass spectrometry. Free Radic. Biol. Med..

[37] Collins A.R. (2002). Inter-laboratory validation of procedures for measuring 8-oxo−7,8-dihydroguanine/8-oxo−7,8-dihydro−2′-deoxyguanosine in DNA. Free Radic. Res..

[38] Ravanat J.L. (2002). Cellular background level of 8-oxo−7,8-dihydro−2’-deoxyguanosine: an isotope based method to evaluate artefactual oxidation of DNA during its extraction and subsequent work-up. Carcinogenesis.

[39] Riis B. (2002). Comparison of Results from different laboratories in measuring 8-oxo−2â€²-deoxyguanosine in synthetic oligonucleotides. Free Radical Research.

[40] Escodd. (2002). Comparative analysis of baseline 8-oxo−7,8-dihydroguanine in mammalian cell DNA, by different methods in different laboratories: an approach to consensus. Carcinogenesis.

[41] Huang X. (2001). Importance of complete DNA digestion in minimizing variability of 8-oxo-dG analyses. Free Radic. Biol. Med..

[42] Escodd (2003). Measurement of DNA oxidation in human cells by chromatographic and enzymic methods. Free Radic. Biol. Med..

[43] Shigenaga M.K. (1994). Assays of oxidative DNA damage biomarkers 8-oxo-2’-deoxyguanosine and 8-oxoguanine in nuclear DNA and biological fluids by high-performance liquid chromatography with electrochemical detection. Methods in Enzymology.

[44] Schwarz K.B. (2001). Increased hepatic oxidative DNA damage in patients with hepatocellular carcinoma. Dig. Dis. Sci..

[45] Jüngst C. (2004). Oxidative damage is increased in human liver tissue adjacent to hepatocellular carcinoma. Hepatology.

[46] Shimoda R. (1994). Increased formation of oxidative DNA damage, 8-Hydroxydeoxyguanosine, in human livers with chronic hepatitis. Cancer Res.

[47] Jaruga P. (1994). Oxidative DNA base damage and antioxidant enzyme activities in human lung cancer. FEBS Lett..

[48] Iida T. (2001). Accumulation of 8-oxo-2’-deoxyguanosine and increased expression of hMTH1 protein in brain tumors. Neuro-oncol..

[49] Klaunig J.E., Kamendulis L.M., Hocevar B.A. (2010). Oxidative stress and oxidative damage in carcinogenesis. Toxicol. Pathol..

[50] Niles J.C., Wishnok J.S., Tannenbaum S.R. (2004). Spiroiminodihydantoin and guanidinohydantoin are the dominant products of 8-oxoguanosine oxidation at low fluxes of peroxynitrite: mechanistic studies with ^18^O. Chem. Res. Toxicol..

[51] Duarte V. (2000). Oxaluric acid as the major product of singlet oxygen-mediated oxidation of 8-Oxo−7,8-dihydroguanine in DNA. J. Am. Chem. Soc..

[52] Müller H., Carell T. (2007). A Carbocyclic Analog of the Oxidatively Generated DNA Lesion Spiroiminodihydantoin. Eur. J. Org. Chem..

[53] White B. (2005). Oxidised guanidinohydantoin (Ghox) and spiroiminodihydantoin (Sp) are major products of iron- and copper-mediated 8-oxo−7,8-dihydroguanine and 8-oxo−7,8-dihydro−29-deoxyguanosine oxidation. Mol. Biosyst..

[54] Dedon P.C. (2007). Challenges in developing DNA and RNA biomarkers of inflammation. Biomark. Med..

[55] Uppu R. (1996). Competitive reactions of peroxynitrite with 2’-deoxyguanosine and 7,8-dihydro−8-oxo−2’-deoxyguanosine (8-oxodG): relevance to the formation of 8-oxodG in DNA exposed to peroxynitrite. Free Radic. Biol. Med..

[56] Tudek B. (2010). Involvement of oxidatively damaged DNA and repair in cancer development and aging. Am. J. Transl. Res..

[57] Croteau D.L., Bohr V.A. (1997). Repair of oxidative damage to nuclear and mitochondrial DNA in mammalian cells. J. Biol. Chem..

[58] Dizdaroglu M. (1991). Chemical determination of free radical-induced damage to DNA. Free Radic. Biol. Med..

[59] Yu H. (2005). Quantitation of four guanine oxidation products from reaction of DNA with varying doses of peroxynitrite. Chem. Res. Toxicol..

[60] Henderson P.T. (2003). The hydantoin lesions formed from oxidation of 7,8-Dihydro-8-oxoguanine are potent sources of replication errors in vivo. Biochemistry.

[61] Mason P.E. (2003). The hydration structure of guanidinium and thiocyanate ions: Implications for protein stability in aqueous solution. Proc. Natl. Acad. Sci. USA.

